# Intraparenchymatous adenomatoid tumor dependent on the rete testis: A case report and review of literature

**DOI:** 10.4103/0970-1591.45551

**Published:** 2009

**Authors:** A. Jiménez Pacheco, J. L. Martínez Torres, F. Valle Díaz de la Guardia, M. A. Arrabal Polo, A. Zuluaga Gómez

**Affiliations:** Urology Service, “San Cecilio” University Hospital, Granada, Spain

**Keywords:** Adenomatoid tumor, diagnosis and ultrasound

## Abstract

The adenomatoid tumor is the most frequent paratesticular tumor. It is a benign tumor, which in women is mainly found in the uterus and the fallopian tubes, while in men it is most frequently found in the epididymis. These lesions may also affect the testicular albuginea, the spermatic cord and, in exceptional cases, the testicular parenchyma, of which there are only 4 published cases, the ejaculatory ducts, prostate, etc. The clinical signs and imaging studies are, on many occasions, difficult to differentiate from malign intratesticular solid tumor, which can result in unnecessary orchidectomies. We present a new case of intraparenchymatous adenomatoid tumor dependent on the rete testis.

## INTRODUCTION

Adenomatoid tumors are those most frequently observed in the paratesticular tissue, representing approximately 32%. In men, the majority are situated in the epididymis but these lesions can also affect the albuginea, the spermatic cord and, in exceptional cases, the testicular parenchyma, the ejaculatory ducts, prostate and suprarenal glands. In women, they mainly affect the fallopian tubes and the uterus.[1]

The classic presentation is between the third and fifth decade of life. The ethnic races most predisposed to this type of tumor are caucasians, followed by blacks (14%) and orientals (0.5%).

In literature, at the moment, only 4 cases of primary intratesticular adenomatoid tumor have been published.[2–5]

## CASE REPORT

A 47 year old patient with no urological history of note complained of experiencing pain in the left testicle for the preceding two months. He had no other symptoms. On examination, a nodule, 1 cm in diameter, in the posterio-superior left testicle could be felt. It was slightly painful and was firm to the touch.

The alpha-fetoproteins and the beta-HCG were normal.

**Figure 1:**
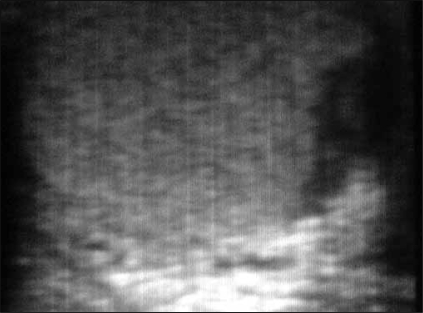
Hypoechoic lesion situated in the superior aspect of the left testicular parenchyma

An ultrasound showed a solid, intratesticular hypoechoic nodular lesion on the left, 8.5 × 7 mm, not encapsulated and without calcification. The epididymis and spermatic cord were normal [Figure 1].

**Figure 2:**
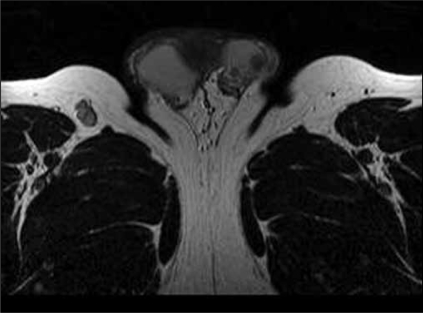
Image in T2 which revealed an intratesticular lesion of 8 mm and a small satellite nodule of 5 mm, hypointense in comparison with the testicular parenchyma. Both were situated in the superior pole of the left testicle.

The lesion could have corresponded to a focal area of inflammation, although other possibilities, such as a tumor of germ cell origin could not be discounted. As a result of the diagnostic uncertainty, a Nuclear Magnetic Resonance scan was performed. This revealed an intratesticular lesion of approximately 8 mm and a small satellite nodule of 5 mm, hypointense in T2 and isointense in T1, in comparison with the testicular parenchyma. Both were situated in the superior pole of the left testicle, adjacent to the head of the epididymis [Figure 2].

As a result of the radiology findings, a left inguinal orchidectomy was performed.

The final anatomopathological diagnosis revealed an off-white regular lesion of 1 × 0.7 cm. It was made up of tubular formations covered with smooth cells, surrounded by fibrous stroma, which had invaded the rete testis. This was compatible with an intraparenchymatous adenomatoid tumor dependent on the rete testis. No other tumor sites were seen in the underlying parenchyma. Diagnostic confirmation was realized through immunohistochemical tests, which were positive to the calretinin markers, CK7 and thromobomoduline.

## DISCUSSION

The term adenomatoid tumor was introduced by Golden and Ash in 1945 to describe a group of benign tumors with glandular pattern, with obscure histogenesis, localized in the urogenital tract.

Although the majority of authors consider it a benign tumor, without good documentation of cases of metastasis or recurrence after excision, there have been some descriptions of malign forms of adenomatoid tumor.[1]

Its origin is controversial. However, the presence of cytoplasmatic keratin, the elevated concentrations of hyaluronic acid and the absence of the carcinoembryonal antigen, related to factor VIII, as well as the presence of the mesothelial antigen, found by using the technique of indirect immunoperoxidase, makes the theory of mesothelial origin, the most accepted in the present situation.[2]

Histologically, the tumor has three basic distinguishing characteristics: tubules, cords, and small clusters covered with cuboid cells, with moderate eosinophilia and vacuolated cytoplasm, the latter being, on occasions, an important diagnostic tool. The stroma, variable in density, is usually fibrous and occasionally hyalinized. On occasion, diagnosis is also helped by the presence of aggregated lymphoids, particularly on the periphery, which are rarely observed in other lesions.

Given its anatomopathological characteristics, problems may occur in the diagnostic differentiation between yolk sac tumor, malign mesothelioma, leiomyoma, Leyding cell tumor and, especially, Sertoli cell tumor, since the tubules of these tumors can be very similar and the vacuoles of adenomatoids can be simulated by lipidized Sertoli cells. Inmunohistochemical confirmation with mesothelial-related markers (calretinin, CK 5/6/7 and WT-1) is helpful in the diferencial with nonmesothelial lesions.

Clinically they present as small lumps, asymptomatic, discovered accidentally, although on occasions, they may be associated with a hydrocele, periorchitis, etc., with markers being negative in all cases.

Pre-operative scrotal ultrasound is the preferred diagnostic method. It differentiates between intra and extratesticular lesions and allows accurate diagnosis of cystic forms and solid lesions, although it cannot determine the benigness of the hypo and hyperechoic, even when the doppler is used.

The adenomatoid tumor does not produce any charactistic patterns on ultrasound which would allow us to distinguish between an intratesticular adenomatoid tumor or a malign intratesticular solid tumor, given that in the literature they can present as hypo, hyper or even isoechoic as regards the adjacent parenchyma. In our case, the Nuclear Magnetic Resonance scan did not allow us to arrive at a definitive diagnosis either.

The value of the intra-operative biopsy has grown in the last few years, as a result of publications which report a significant prevalence of benign testicular lesions (10-20% of the neoplastic testicular tumors).

Accordingly, when a benign lesion is suspected, it is recommended that an inguinal exploration and intraoperative biopsy be carried out, prior to clamping of the spermatic cord. If the benign nature of the lesion is confirmed, it is sufficient to perform a tumorectomy with wide margins of resection, in this way avoiding an orchidectomy.

Follow up of these patients with testicular ultrasound is enough to show any relapse and, in our case, none has been documented to date.

To date, only four cases of primary intratesticular adenomatoid tumor have been published,[2–5] Hostman et al.[2] being the first to publish in 1992. Only in the case, published by Samad et al.[4] was a tumorectomy carried out, so conserving the testicle.

In conclusion, the adenomatoid tumor is a benign lesion with clinical signs similar to those of malign testicular neoplasm. The radiographic findings are non-specific, presenting as iso, hypo, or hyperechoic nodules, which do not allow us, in most cases, to differentiate between a benign or malign solid intraparenchymatous lesion. Nor does the Nuclear Magnetic Resonance scan allow conclusive diagnosis. This explains, except in one case published, in the other orchidectomy was performed.

Progress is favorable in all cases, with no cases of relapse published, even when the margins of resection have not been wide, for which follow-up by post-operative ultrasound is sufficient in these patients.
